# Amelioration of Oxidative Stress in Rats with Chronic Obstructive Pulmonary Disease through Shenqi Huatan Decoction Activation of Peroxisome Proliferator-Activated Receptor Gamma-Mediated Activated Protein Kinase/Forkhead Transcription Factor O3a Signaling Pathway

**DOI:** 10.1155/2024/5647813

**Published:** 2024-07-02

**Authors:** Jingjing Chen, Wenxiao Qiao, Xiaoming Xue, Dian Li, Ye Zhang, Di Xie, Jinyun Wang, Yaoqin Sun, Shuo Yang, Zhuomin Yang

**Affiliations:** ^1^ Department of Respiration Shanxi Province Hospital of Traditional Chinese Medicine, Taiyuan 030012, China; ^2^ Department of Respiration Institute of Shanxi Traditional Chinese Medicine, Taiyuan 030012, China; ^3^ Shanxi University of Traditional Chinese Medicine, Taiyuan 030619, China

## Abstract

**Background:**

Chronic obstructive pulmonary disease (COPD) is a common respiratory disease. Currently, no specific treatment strategy has been established; therefore, finding new treatment methods is essential. Clinically, Shenqi Huatan Decoction (SQHT) is a traditional Chinese medicinal formula for COPD treatment; however, its mechanism of action in treatment needs to be clarified.

**Methods:**

The COPD rat model was replicated by cigarette smoking and tracheal injection using the LPS method. The control group and the SQHT groups were treated with dexamethasone and SQHT by gavage, respectively. After treatment, superoxide dismutase (SOD) serum levels, total antioxidant capacity (TAOC), lipid peroxidation, and malondialdehyde (MDA) were detected by enzyme-linked immunosorbent assay (ELISA). Activated protein kinase alpha (AMPK-*α*), forkhead transcription factor O3a (FOXO3a), manganese SOD (MnSOD), and peroxisome proliferator-activated receptor gamma (PPAR*γ*) were detected using reverse transcriptase quantitative polymerase chain reaction (RT-qPCR) and Western blot. Microribonucleic acid and protein expression levels were measured, and pathological changes in lung tissue were observed using hematoxylin and eosin staining.

**Results:**

The pathological findings suggested that SQHT substantially affects COPD treatment by enhancing alveolar fusion and reducing emphysema. ELISA results showed that SQHT could lower the blood levels of MDA and lipid peroxide and raise SOD and TAOC levels, suggesting that it could lessen oxidative stress. In the lung tissue of rats with COPD, large doses of SQHT intervention dramatically increased AMPK protein expression, AMPK-*α*, FOXO3a, MnSOD, and PPAR*γ*, indicating that SQHT may reduce oxidative stress by activating the PPAR*γ*-mediated AMPK/FOXO3a signaling pathway. Similar results were obtained using RT-qPCR.

**Conclusion:**

SQHT is effective for COPD treatment. The mechanism of action may be related to the activation of the PPAR*γ*-mediated AMPK/FOXO3a signaling pathway to improve oxidative stress in lung tissue.

## 1. Introduction

Chronic obstructive pulmonary disease (COPD) accounts for a high proportion of cases of respiratory diseases, with a high incidence of morbidity and mortality. It is also associated with a high risk of cardiovascular disease, which threatens patients' quality of life and health [1]. The high morbidity, disability, and mortality rates associated with COPD make it a public health issue that must be addressed. Oxidative stress plays a crucial role in the development of COPD [2]. Smoking is a significant risk factor for COPD. Tobacco smoke contains high levels of oxygen radicals and reactive oxygen species (ROS), which cause airway inflammation, leading to membrane lipid peroxidation and structural and functional alterations in various biomolecules, resulting in an imbalance between oxidation and antioxidation [3]. Regulation of the intracellular oxidation/antioxidation balance is essential for COPD prevention and treatment [4]. Under physiological conditions, the body's oxidants and antioxidants exist in a dynamic balance; oxidative stress occurs when an imbalance leads to cell death and tissue damage. Owing to their unique tissue structure, rich blood supply, and large respiratory membrane area, the lungs are highly susceptible to damage by endogenous oxidants such as ROS, reactive nitrogen species, and lipid peroxidation products.

Traditional Chinese medicine is a rich theoretical system for treating COPD. This system alleviates patients' clinical symptoms and effectively prevents the acute onset of COPD. The search for corresponding target interventions from traditional Chinese medicine for COPD is of great practical significance. Traditional Chinese medicine plays an irreplaceable role in prevention and treatment due to its abundant resources, low economic cost, precise therapeutic effect, seeking the root cause of the disease, and considering both specimens. The traditional Chinese medicine, Shenqi Huatan Decoction (SQHT), is a combined formula of Shenqi Chongcao capsules and phlegm lowering capsules, consisting of American ginseng, Panax ginseng, Cordyceps, Perillae, white mustard seed, Bai Qian, Golden Boiling Herb, and roasted Ephedra. It has long been used for treating COPD by Professor Han Mingxiang, a master of Chinese medicine, and has definite results. Previous studies have shown that it improves airway remodeling and reduces airway inflation by invading transforming growth factor beta-simulated signaling paths in active sites of modeling and clustering [5]. This study explored the potential mechanism of action of SQHT in reducing oxidative stress in rats with COPD by activating the peroxisome proliferator-activated receptor gamma (PPAR*γ*)-mediated activated protein kinase (AMPK)/forkhead transcription factor O3a (FOXO3a) signaling pathway at the cellular level.

## 2. Materials and Methods

### 2.1. Animals

**Figure 1:**
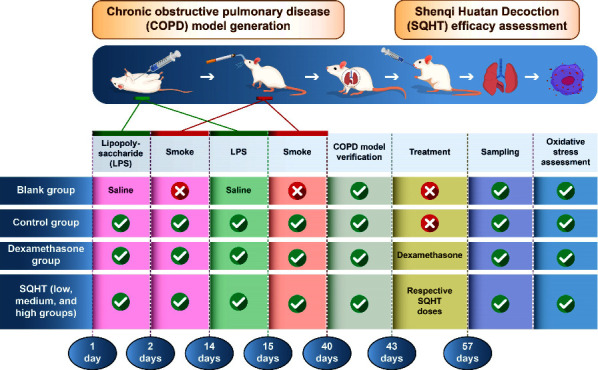
Experimental flowchart: chemicals/reagents.

In total, 60 healthy male Sprague–Dawley rats with a specific pathogen-free rating, aged 4–6 weeks, and weighing an average of 120 ± 10 g were bought from Beijing Huafukang Laboratory Animal Co. (China). Using the random number table method, the 60 rats were randomly divided into six groups of 10 rats each. The groups are blank, model, dexamethasone, and SQHT high-, medium-, and low-dose. In the blank group, physiological saline was injected without medication and gavage. No medication was administered after modeling in the model group, and physiological saline was administered by gavage. In the dexamethasone group, dexamethasone tablets were administered after modeling. In the low-dose SQHT group, after modeling, the low-dose Shenqi Huatan formula was administered orally. In the middle-dose SQHT group, after modeling, a middle dose of the Shenqi Huatan formula was administered by gavage. In the high-dose SQHT group, after modeling, the high-dose Shenqi Huatan formula was administered orally. The experimental design is shown in Figure 1.

SQHT was composed of Radix Rhizoma ginseng (10 g), Panax notoginseng (3 g), Perilla frutescens (10 g), Sinapis semen (10 g), Flos Inulae Japonicae (8 g), Golden Boiling Herb (8 g), Cordyceps sinensis (5 g), and roasted Ephedra (6 g). All herbs were provided by the Shanxi Provincial Hospital of Traditional Chinese Medicine (Shanxi, China). The drug was then steeped for 30 min, boiled, and filtered to obtain the initial liquid. After boiling twice, the two solutions were combined, filtered, and concentrated to 15 mg/mL.

The reagents used include lipopolysaccharide (LPS, Sigma, L2880), dexamethasone (Tianjin Lisheng Pharmaceutical Co., Ltd, 2111003), cigarette (Yunyun, China), HiFiScript rapid genomic complementary deoxyribonucleic acid (cDNA) first-strand removal synthesis kit (Conway Reagents, Cat# CW2582M), fluorescent quantitative polymerase chain reaction (qPCR) kit (Mona Bio, Cat# MQ00401S), superoxide dismutase (SOD), enzyme-linked immunosorbent assay (ELISA) kit (Annoroad Genome Technology Co., Ltd, Cat# GR20221110), total antioxidant capacity (TAOC) ELISA kit (Annoroad Genome Technology Co., Ltd, Cat# GR20221110), lipid peroxide (LPO) ELISA kit (Annoroad Genome Technology Co., Ltd, Cat# GR20221110), malondialdehyde (MDA) ELISA kit (Annoroad Genome Technology Co., Ltd, Cat# GR20221110), the adenylate-AMPK antibody (Abcam, Cat# ab32047), FOXO3a antibody (Abcam,Cat# ab109629), phosphorylated-FOXO3a (p-FOXO3a) antibody (Abcam,Cat# ab26649), glyceraldehyde-3-phosphate dehydrogenase (GAPDH) antibody (Abcam, Cat# ab9485), manganese SOD (MnSOD) antibody (Beyotime Biotech. Inc., Cat# AF5144), and PPAR*γ* antibodies (Bioss, bs-0530R).

### 2.2. Induction of COPD in Rats

The Experimental Animal Ethics Committee of the Institute of Shanxi Traditional Chinese Medicine in Taiyuan, China, approved this study (no. SZYLY2022KY-0514). All methods were performed in accordance with the relevant regulations and animal research: Reporting of *In Vivo* Experiment Guidelines. The animals were allowed to adapt to the environment for one week before starting the experiment.

The COPD rat model was replicated by cigarette smoking and tracheal injection using the LPS method [6]. LPS was injected into the trachea of rats on days 1 and 14 at a concentration of 0.2 ml/rat in the blank group. Rats in the blank group were administered a similar volume of 0.9% saline. On days 2–13 and 15–40, rats passively smoked 10 cigarettes per day for 40 min.

### 2.3. Drug Administration

Dosing was started at the end of the modeling and administered once daily for 14 days.

- The blank and model groups were administered 0.9% sodium chloride solution by oral gavage.
- The dexamethasone group was gavaged with a dexamethasone suspension using an equivalent dose conversion formula of 0.75 mg/60 kg  × 7.5 = 0.09 mg/kg daily for rats.
- In the treatment group, rats were administered SQHT by gavage. The daily doses of the drug in the high-, medium-, and low-dose groups were 15, 7.5, and 3.8 times the clinical dose, respectively. The daily drug doses were 8.6 g/kg, 4.3 g/kg, and 2.2 g/kg for the high-, medium-, and low-dose groups, respectively.

### 2.4. Hematoxylin and Eosin Staining to Observe Histopathological Changes in Rat Lungs

After the rats were anesthetized with 10% pentobarbital sodium, the skin of the chest and abdomen were cleaned with 75% ethanol, and the muscles from the abdomen to the neck were cut under sterile conditions. Blood was withdrawn from the inferior vena cava and both lungs were removed. The right lung was immediately frozen in an ultralow-temperature refrigerator at −80°C for testing. The left lung was fixed in 4% paraformaldehyde solution, embedded in paraffin, sectioned, and stained with H&E stain. The structural changes in the alveoli and airway walls were observed under a microscope.

### 2.5. SOD, TAOC, LPO, and MDA Levels in Rat Serum Were Measured by ELISA

After blood was collected from the inferior vena cava, it was centrifuged using a high-speed centrifuge at 1500 rpm for 20 min, and the supernatant was collected and tested in strict accordance with the ELISA kit instructions.

The mRNA expression of AMPK-*α*, FOXO3a, MnSOD, and PPAR*γ* was detected using real-time fluorescence quantitative PCR

The total ribonucleic acid (RNA) from lung tissues was extracted by the TRIzol method, incubated at 42°C for 15 min and 85°C for 5 min, and reverse transcribed to cDNA. PCR amplification was performed using this template. The reaction conditions were as follows: step 1, predenaturation at 95°C for 15 min, and step 2, 95°C for 20 s, 55°C for 30 s, and 72°C for 30 s, 40 cycles. Primers were designed and synthesized by Sangon Biotech Co., Ltd. (Shanghai, China), and the specific sequences are listed in Table 1. The relative quantification of each indicator microRNA was carried out using the 2^−△△CT^ method with *β*--actin as the internal reference gene.

### 2.6. Protein Expression of AMPK-*α*, FOXO3a, p-FOXO3a, MnSOD, and PPAR*γ* Was Detected by Western Blot

Lung tissues were lysed in a radioimmunoprecipitation assay buffer. The lysate was centrifuged at 12000 rpm for 15 min at 4°C, and the supernatant was collected for further analysis. The total protein concentration was determined using a Bicinchoninic acid protein assay kit. Following electrophoresis and transfer, the membrane was blocked with 5% nonfat dry milk for 1 h at room temperature. The primary antibody was diluted as instructed, and the membrane was incubated with primary antibodies overnight at 4°C. After washing with Tris-buffered saline with 0.1% Tween 20 detergent, the membranes were incubated with appropriate horseradish peroxidase-conjugated secondary antibodies for 2 h at room temperature. The polyvinylidene fluoride membranes were then incubated with chemiluminescent reagents to visualize the bands. The relative expression of the target protein was expressed as the ratio of the target protein's gray value to the internal reference, using GAPDH as the internal reference. ImageJ software was used to detect the gray values of the western blot bands.

### 2.7. Statistical Analysis

Statistical analysis was performed using SPSS 26.0 and GraphPad Prism 8.0. The experimental data are all measures expressed as mean ± standard deviation. If the distribution was normal, one-way analysis of variance was chosen as the statistical method, and if the variance was the same, Tukey's post hoc analysis was chosen for multiple comparisons between the two groups. *P* < 0.05 indicated statistically significant differences, whereas *P* < 0.01 indicated highly statistically significant differences.

## 3. Results

### 3.1. Histopathological Sections of the Lung

**Figure 2:**
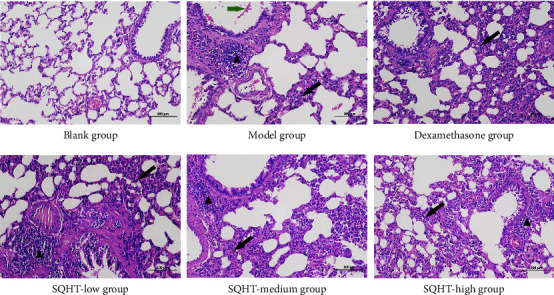
Effects of Shenqi Huatan Decoction (SQHT) and dexamethasone on histological changes in hematoxylin and eosin (H&E)-stained lung sections of chronic obstructive pulmonary disease (COPD) rats. The black arrow indicates extensive infiltration by granulocytes and macrophages. The green arrow symbolizes multifocal hemorrhaging. The blank triangle represents local lymphocyte infiltration around blood vessels and trachea.

The alveolar septum between adjacent alveoli in the blank group is relatively thin, with clear boundaries, complete alveolar wall structure, no fusion between alveoli, and no obvious abnormalities. In the model group, alveolar consolidation, alveolar fusion, widened septa, development of pulmonary bullae, infiltration of a large number of granulocytes and macrophages in the airway and between the alveoli, and scattered distribution of a small number of granulocytes, macrophages, and foam cells in the surrounding alveoli can be seen. At the same time, bleeding was present in the small bronchi. Both the control group and the low-dose, medium-dose, and high-dose SQHT groups showed varying degrees of alveolar enlargement, partial alveolar fusion, uneven infiltration of inflammatory cells around the tracheal wall, partial shedding of tracheal mucosal epithelium, and varying degrees of narrowing of the lumen. After treatment, the alveolar status in the SQHT groups and the dexamethasone group improved compared to the model group, with reduced infiltration of inflammatory cells and no bronchial bleeding observed. The SQHT high-dose group and the dexamethasone group had the best state (Figure 2). Based on *the INHAND Proposal (International Harmonization of Nomenclature and Diagnostic Criteria for Lesions in Rats and Mice)* [7], three samples from each group were selected for pathological scoring, with the specific results listed in Table 2.

### 3.2. SQHT Can Increase the Content of SOD and TAOC and Reduce the Content of LPO and MDA

**Figure 3:**
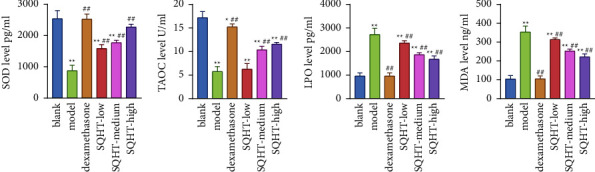
Effect of SQHT and DEX on the expression of SOD, TAOC, LPO, and MDA in serum from chronic obstructive pulmonary disease rats. Compared with the blank group, ^*∗*^*P* < 0.05 and ^*∗∗*^*P* < 0.01; compared with the model group, ^#^*P* < 0.05 and ^##^*P* < 0.01.

Compared with the blank group, serum SOD and TAOC in the rats of the model group were significantly reduced, and MDA and LPO were significantly increased (*P* < 0.01). Compared with the model group, MDA and LPO were significantly lower, and SOD and TAOC were significantly higher in each treatment group (*P* < 0.01), with the most significant difference in the high-dose group (Figure 3).

### 3.3. SQHT Significantly Increased the mRNA Expression of AMPK-*α*, FOXO3a, MnSOD, and PPAR*γ*

**Figure 4:**
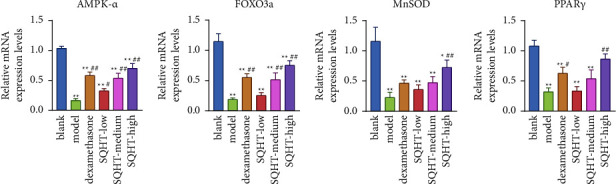
Effect of SQHT and DEX on the expression levels of AMPK-*α*, FOXO3a, MnSOD, and PPAR*γ* in lung tissues of COPD rats. Compared with the blank group, ^*∗*^*P* < 0.05 and ^*∗∗*^*P* < 0.01; compared with the model group, ^#^*P* < 0.05 and ^##^*P* < 0.01.

The lung tissue of rats in the model group had significantly lower mRNA expression levels of AMPK-*α*, FOXO3a, MnSOD, and PPAR*γ* than the blank group (*P* < 0.01). In comparison with the model group, the control and SQHT medium- and high-dose groups had significantly increased mRNA expression of AMPK-*α* and FOXO3a (*P* < 0.01), the high-dose group only had significantly increased mRNA expression of MnSOD (*P* < 0.01), and the control and high-dose groups had significantly increased mRNA expression of PPAR*γ* (*P* < 0.05 and *P* < 0.01, respectively) (Figure 4).

### 3.4. SQHT Significantly Increased the Protein Expression Levels of AMPK-*α*, FOXO3a, p-FOXO3a, MnSOD, and PPAR*γ*

**Figure 5:**
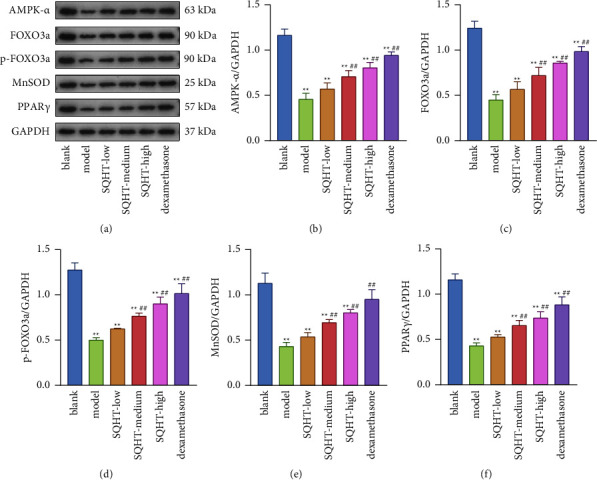
Effect of SQHT and DEX on the protein expression levels of AMPK-*α*, FOXO3a, p-FOXO3a, MnSOD, and PPAR*γ* in lung tissues of COPD rats. GAPDH was used as the internal control, representative western blot of AMPK-*α*, FOXO3a, p-FOXO3a, MnSOD, and PPAR*γ* (a), and the quantitative analysis (b‐f). Compared with the blank group, ^*∗*^*P* < 0.05 and ^*∗∗*^*P* < 0.01; compared with the model group, ^#^*P* < 0.05 and ^##^*P* < 0.01.

Compared with the blank group, the protein expression levels of AMPK-*α*, FOXO3a, p-FOXO3a, MnSOD, and PPAR*γ* were significantly lower in the lung tissues of rats in the model group (*P* < 0.01). Compared with the model group, the abovementioned indices were significantly higher in the control and SQHT medium- and high-dose groups (*P* < 0.01), with the most significant difference in the control group (Figure 5).

## 4. Discussion

Oxidative stress plays a vital role in COPD pathogenesis and is mainly caused by the accumulation of endogenous and exogenous oxidants. The primary sources of exogenous ROS are cigarette smoke and airborne particulate matter [8]. High levels of free radicals and other oxidants in cigarette smoke dissolve in the fluid that lines the airway epithelium upon entry into the airways, accumulate in the lungs, irritate the lung tissue over time, sustain inflammation, and aid in developing COPD [9]. The inflammatory response and hypoxia are essential factors in producing endogenous ROS. An increase in either endogenous or exogenous ROS disrupts the oxidative/antioxidative balance, activating the pulmonary and systemic oxidative stress response in patients with COPD. Regulating oxidative stress is highly significant in COPD prevention and treatment.

During oxidative stress in COPD, ROS causes airway epithelial cell damage, induces airway smooth muscle cell proliferation, promotes airway wall thickening, and increases the rate of decline in lung function [10]. An essential indicator of oxidative stress in COPD, the TAOC, might represent the antioxidant status [11]. SOD is an endogenous antioxidant and metalloactive enzyme that scavenges oxygen radicals. It provides insights into the body's free radical metabolism and is crucial for maintaining a proper balance between oxidative stress and antioxidant defenses [12]. Studies have shown that airway inflammation in patients with COPD can lead to impaired oxygen exchange, resulting in ischemia and hypoxia in the lungs and reduced SOD activity [13]. Oxidants readily oxidize lipids and lipid peroxidation is a major consequence of oxidative stress. LPO damages vascular endothelial cells, alters biological membrane structure, increases the number of leukocytes, promotes platelet chemotaxis and aggregation, and promotes free radicals production, thereby inhibiting the body's immune response [14]. Enhanced peroxidation in the body increases the level of LPO, which reduces its ability to scavenge oxygen radicals, thereby reducing SOD activity and causing abnormalities in cell structure [15].

MDA is one of the end products of peroxidation reactions, which can indirectly represent the severity of cellular attack by free radicals [16], and is a marker of lipid peroxidation. It can cross-link macromolecules in organisms, including proteins, nucleic acids, and lipids, leading to biofilm degradation and cell death due to reactions between ROS and unsaturated fatty acids. The results of this study showed that SOD and TAOC levels were significantly lower and MDA and LPO levels were significantly higher in the serum of COPD rats. Following therapy, the levels of MDA and LPO were significantly lower and those of SOD and TAOC were significantly higher. The therapeutic effects of SQHT and dexamethasone were demonstrated by improved oxidative stress and enhanced antioxidant capacity in rats with COPD.

PPAR*γ* belongs to the superfamily of ligand-activated transcription factors, which recognize lipid oxidation products such as oxidized phospholipids and nitroolefins to sense oxidative stress and inhibit oxidative stress by regulating oxidase activity [17]. By activating several pathways to limit the body's exposure to free radicals and by competitively suppressing the inflammatory mediators produced by associated inflammatory signaling pathways, PPAR is a crucial transcription factor in the body's inflammatory response and oxidative stress [18].

The adenylate-AMPK/FOXO3a pathway plays a vital role in reducing ROS accumulation of reactive oxygen species and combating oxidative and inflammatory responses [19]. AMPK, a crucial cellular protein kinase present in cells, regulates energy and metabolism in the body. This process requires intricate signaling pathways and regulatory processes. It has been found that ROS produced by mitochondria can act as a signaling molecule to activate AMPK [20], which suggests that AMPK is redox-sensitive. AMPK activation reduces ROS accumulation and protects fibroblasts against oxidative stress damage [21]. FOXO3a is a member of the transcription factor family that is important for cell proliferation, apoptosis, and oxidative stress [22]. As a direct downstream target of AMPK, FOXO3a is nucleated when AMPK is activated, which increases the activity of ROS-detoxifying enzymes, reduces ROS-induced stress, and aids in cell survival. Catalase (Cat) and MnSOD are direct targets of FoxO3a. Therefore, increased levels of Cat and MnSOD following FoxO3a activation could effectively manage ROS levels and reduce ROS-induced stress [23]. MnSOD, a form of SOD, is an essential endogenous antioxidant protein and a member of the metalloantioxidant enzyme family. AMPK activation has also been reported to upregulate MnSOD levels [24]. PPAR*γ* plays an essential biological function in cell differentiation and metabolic regulation. By regulating downstream forkhead proteins, PPAR*γ* can phosphorylate AMPK, thereby activating the AMPK/FOXO3a signaling pathway and exerting antioxidant effects [25]. Wang [26] found that PPAR*γ* significantly increased AMPK activity and protected airway epithelial cells from inflammatory damage.

In accordance with the results of our investigation, PPAR*γ* markedly boosted AMPK activation and shielded airway epithelial cells from inflammatory damage. In this experiment, the protein expression levels of AMPK-*α*, FOXO3a, p-FOXO3a, MnSOD, and PPAR*γ* in the lung tissues of the COPD model group were significantly lower than those of the blank group. In contrast, they were significantly higher compared to the model group and in the control group and the SQHT medium-dose and high-dose groups. Therefore, it can be concluded that the activation of PPAR*γ* can enhance the expression of AMPK, which can induce the nucleation of FOXO3a and upregulate the phosphorylation level of FOXO3a. FOXO3a binds to the antioxidant gene MnSOD, upregulates the levels of antioxidant enzymes, scavenges excess oxygen radicals, and reduces oxidative damage.

## 5. Conclusions

This experiment showed that treatment of COPD rats with SQHT improved the histopathological changes in the lungs with significant efficacy. The mechanism may be related to the activation of the AMPK/FOXO3a signaling pathway by PPAR*γ* to reduce oxidative stress in the lungs of rats.

## Figures and Tables

**Table 1:** Primer sequences for target genes.

Name	Gene name	GenBank accession	Gene ID	Serials	Length of output (bp)
AMPK-*α*	Prkaa1	NM_019142	65248	GCCAAATCAGGGACTGCTAC	132
				GGAGGTCACGGATGAGGTAA	

FOXO3a	FOXO3	NM_001106395	294515	ACCTGTCCTACGCTGACCTGA	163
				TTGTGGCGGATGGAGTTCTT	

MnSOD	Sod2	NM_017051	24787	AGCCTCCCTGACCTGCCTTAC	138
				CGCCTCGTGGTACTTCTCCTC	

PPAR*γ*	PPARG	NM_013124	25664	CCTTTACCACGGTTGATTTCTC	139
				GGCTCTACTTTGATCGCACTTT	

*β*-actin	Actb	NM_031144	81822	AGATTACTGCCCTGGCTCCTAG	144
				CATCGTACTCCTGCTTGCTGAT	

**Table 2:** Pathological scores (lung tissue).

	Inflammatory cell infiltration	Thickening of the alveolar wall	Bleeding	Congested blood	Total score
Blank	0	0	0	0	0
Model	2	2	2	0	6
Dexamethasone	1	2	0	0	3
SQHT low-dose	2	2	0	0	4
SQHT medium-dose	2	2	0	0	4
SQHT high-dose	2	1	0	0	3

The higher the total score, the more severe the pathological damage, while the lower the score, the less severe the damage.

## Data Availability

The data used to support the findings of this study are available from the corresponding author upon request.
